# Electrocatalysis of Oxygen Evolution Reaction Promoted by CoNiMn Films Synthesized by Electrodeposition

**DOI:** 10.1021/acsomega.4c05057

**Published:** 2024-10-17

**Authors:** Ana Luisa Silva, Marcos V. Colaço, Liying Liu, Yutao Xing, Nakédia M. F. Carvalho

**Affiliations:** † Universidade do Estado do Rio de Janeiro (UERJ), Instituto de Química, Rua São Francisco Xavier, 524, Rio de Janeiro, 20550-900 Rio de Janeiro, Brasil; ‡ Universidade do Estado do Rio de Janeiro (UERJ), Instituto de Física, Rua São Francisco Xavier, 524, Rio de Janeiro, 20550-013 Rio de Janeiro, Brasil; § Centro Brasileiro de Pesquisas Físicas (CBPF), Rua Doutor Xavier Sigaud 150, Rio de Janeiro, 22290180 Rio de Janeiro, Brasil; ∥ Universidade Federal Fluminense, Instituto de Física, Niterói, 24210-346 Rio de Janeiro, Brasil

## Abstract

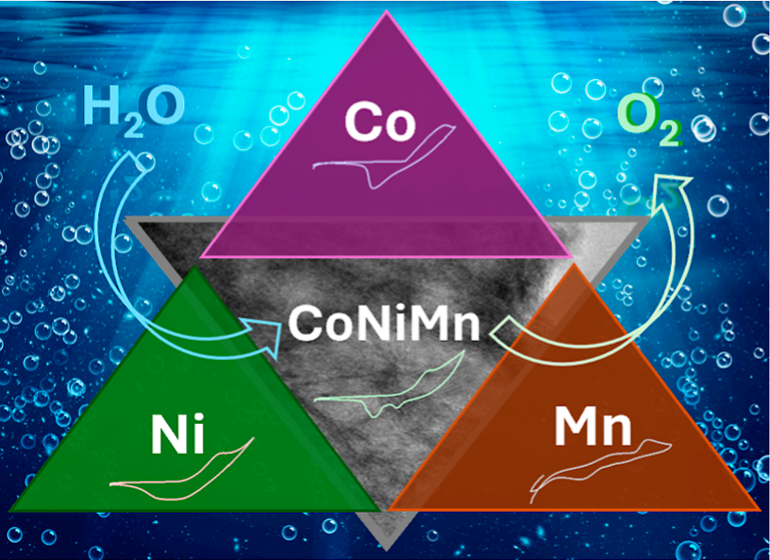

Recently, efforts have been made to address the environmental damage caused by fossil-fuel-based primary energy sources. Interest in efficient technologies for converting and storing energy using renewable sources, especially sunlight, has increased, with the aim of replicating the natural photosynthesis process. However, artificial photosynthesis faces challenges with unfavorable kinetics and thermodynamics, requiring the use of stable catalysts for the hydrogen evolution (HER) and oxygen evolution (OER) reactions to generate H_2_ and O_2_, respectively. OER is the most prohibitive of the half-reactions by the highly sluggish kinetics. Mixed oxides, particularly those based on first-row transition metals, have shown promising results as catalysts for the OER. This work reports the synthesis of CoNiMn oxide via electrodeposition on fluoride tin oxide followed by electrochemical activation. This approach seeks to explore the synergistic effect between the elements and to produce a catalyst with superior efficiency and stability for the electrocatalysis of the OER compared to the monometallic and bimetallic oxides. The CoNiMn film was structurally and electrochemically characterized. The electrodeposited CoNiMn hybrid films demonstrated low overpotentials compared with standard OER electrocatalysts, with CoNiMn films outperforming all single and bimetallic oxide films. The activated CoNiMn film required an overpotential of 100 mV at 10 mA cm^–2^ (430 mV at 25 mA cm^–2^) and Tafel slope of 58 mV dec^–1^. The film was active for 15 h at 100 mA cm^–2^ and showed no significant change in morphology and structure after the chronopotentiometry, indicating that it is a promising and cost-effective alternative to enhance the OER activity using abundant elements.

## 1 Introduction

Planet Earth has recently reached the mark of 8 billion people, and to meet the energy demand of this population, sustainable energy sources that harm the environment as little as possible are needed.^1^ Among the various options for sustainable energy development, the production of green hydrogen as a substitute for fossil fuels has stood out.^2,3^ Water electrolysis is one of the most attractive processes for the generation of green hydrogen;^4−6^ however, it is thermodynamically unfavorable and has slow kinetics, requiring efficient catalysts to enable it.^7−9^

Currently, precious-metal-based electrocatalysts (e.g., Ir, Ru, Pt, and Pd) have been demonstrated to be the most active in promoting the oxygen evolution reaction (OER); however, the high cost and scarcity hinder their applications on a large scale. In this concern, many oxides or hydroxides based on first-row transition metals have been described as efficient electrocatalysts because of their stability in strongly oxidizing and alkaline electrolytes, their rich redox properties, lower price and toxicity, and Earth-abundance.^3,10,11^

Many approaches are described to improve the OER activity, and one that has recently gained great attention is the combination of different elements. In this sense, trimetallic electrocatalysts have shown superior efficiency for OER over the bimetallic and monometallic analogues.^12−21^ Trimetallic materials based on first-row transition metals like Mn–Co–Ni oxides have emerged as promising electrocatalysts for OER as reported in recent works such as MnCoNiOx,^20,22−25^ Mn–Ni–Co–P,^26−28^ Ni–Co–Mn oxides,^21^ and Mn/Co/Ni metal–organic framework.^29,30^ Overall, the use of trimetallic catalysts in the water separation process can lead to improved efficiency and sustainability, making them a promising option for green hydrogen production.^31−34^

Substrates such as fluoride tin oxide (FTO), nickel foam, or glassy carbon are usually used as modified electrodes in combination with mixed metal oxides catalysts. According to Fan et al., the deposition method has a great effect on electrocatalyst efficiency. Substrate/electrode like FTO forms a dual-layer structure hampering the transport of electrons from the film to the substrate, slowing the OER. Oppositely, the deposit of the catalyst composed of multimetal oxide components grown by electrodeposition can improve the catalytic performance toward OER, as well as the stability and homogeneity of the film on the substrate/electrode interface in comparison to the dropping cast deposition, for instance.^35^

Activation is another factor being considered to improve the activity of transition metal-based oxides as OER catalysts.^36−38^ Activation processes induce structural changes on the surface of the films by altering the electronic structure through oxide deformation.^39^ Modifying the electronic structure of the oxide by varying the strain changes the formation of oxygen vacancies and migration energetics.^39,40^ OER catalyst activations are generally performed by applying high positive potentials, promoting the formation of more oxidized species.^40,41^ However, using cyclic voltammetry (CV) for activation increases the deformation of the oxides, forming species with different mixed electronic structures, particularly in mixed oxides.^40,41^

Herein, we report the synthesis of CoNiMn oxide by electrodeposition over FTO, where we explored an electrochemical activation of the prepared film and evaluated the effect on the efficiency and stability toward the OER electrocatalysis, in comparison with the Mn, Ni, and Co monometallic and bimetallic oxides. This approach seeks to explore the synergistic effect between Mn, Ni, and Co and to produce a catalyst with superior activity and stability for OER electrocatalysis compared with the monometallic oxides. The CoNiMn films were characterized by powder X-ray diffraction (XRD). The morphology was examined by scanning electron microscopy (SEM), transmission electron microscopy (TEM), and atomic force microscopy (AFM). Electrochemical characterization was carried out by CV, electrochemical impedance spectroscopy (EIS), and electrochemical active surface area (ECSA).

## 2 Experimental Section

### 2.1 Materials

The analytical-grade chemicals and reagents were used as received: Co(NO_3_)_2_·4H_2_O ≥ 98.0%, Mn(NO_3_)_2_·4H_2_O ≥ 97.0%, Ni(NO_3_)_2_·6H_2_O ≥ 98.5%, KOH ≥ 85.0%, and FTO glass plates with 7 Ω per square surface resistivity were purchased from Sigma-Aldrich; ethyl alcohol > 99.0% and acetone > 99.0% from Tedia. FTO glass plates were previously cut into slides of 1 cm × 3.5 cm (W × H). Before deposition, the FTO slides were first sonicated in soap water, then in ethanol and acetone for 10 min, and finally rinsed with deionized water (DI water).

### 2.2 Electrodeposition of the Films

The synthesis of the working electrodes (WEs) by electrodeposition of the CoNiMn mixed oxide was conducted in a solution containing Mn(NO_3_)_2_·4H_2_O (0.1 mol L^–1^), Co(NO_3_)_2_·4H_2_O (0.1 mol L^–1^), Ni(NO_3_)_2_·4H_2_O (0.1 mol L^–1^), and KCl (0.1 mol L^–1^) prepared in DI water and ultrasonicated for 10 min at room temperature. The electrodeposition was carried out in a constant potential mode, applying a potential of −1.10 V for 200 s. After electrodeposition, all films were dried at room temperature in a desiccator for 24 h. After that, the films were electrochemically activated through a single scan in CV varying the potential from 0 to 1.6 V (versus RHE) at KOH (1 mol L^–1^) and scan rate of 0.1 V s^–1^. Finally, the films were characterized and tested in the OER electrocatalysis.

The modification of the FTO with the single metal oxides, Mn, Co, and Ni, and bimetallic oxides, NiMn, CoMn, and CoNi, was proceeded in the same way as with the trimetallic mixed oxide; however, the solutions consisted of KCl (0.1 mol L^–1^) prepared in DI water and the respective salt or respective combination of the salts: Mn(NO_3_)_2_·4H_2_O (0.1 mol L^–1^), Ni(NO_3_)_2_·4H_2_O (0.1 mol L^–1^), or Co(NO_3_)_2_·4H_2_O (0.1 mol L^–1^).

### 2.3 Characterization of the Films

The crystalline structure of the samples was examined by XRD (Bruker D8 ADVANCE) patterns of electrodeposited CoNiMn films (5–80°, step size of 0.02°, and scan rate of 0.04°/min) using Cu Kα radiation. SEM (SEM-FEG JEOL JSM-7100 F microscope, JEOL, Japan) equipped with a field emission gun was used to analyze the morphology of the samples. The TEM and scanning transmission electron microscopy images were acquired on a JEOL JEM2100F instrument to analyze the morphology of the samples. AFM images of the catalyst films were obtained on a Park NX10 (Park, Korea). Experiments were conducted using a 2.8 N/m Pt/Ir probe in intermittent contact (topography, phase contrast, and electrical images). Kelvin force and capacitance coupling measurements were conducted in parallel by applying an electric AC signal at 10 kHz to the metal-coated cantilever. Digested samples were analyzed using an inductively coupled plasma optical emission spectrometry (ICP-OES) model iCAP 6000 series (Thermo Scientific) equipped with a Babington V-Groove type nebulizer (SCP Science, Canada) and a cyclonic spray chamber. Argon with a minimum purity of 99.95% was used as the main, auxiliary, and nebulizer gas. The operational parameters for all measurements: radiofrequency power of 1300 W, main gas flow rate of 15.00 L min^–1^, auxiliary gas rate of 1.00 L min^–1^, and nebulizer gas flow rate of 0.39 L min^–1^.

### 2.4 Electrochemical Measurements

All electrochemical experiments were performed in triplicate using an Autolab PGSTAT302N potentiostat/galvanostat (Metrohm, Switzerland), controlled by the NOVA software (Metrohm). The electrochemical system consisted of a 30 mL three-electrode cell, an Ag|AgCl electrode (3 mol L^–1^ KCl) was used as the reference electrode, a platinum bar as the counter electrode, and the FTO glass plates modified with MnCoNi, monometallic, or bimetallic electrodeposited films used as the WE. Experiments of CV were carried out at 50 mV s^–1^ varying the potential from 0.6 to 1.8 V (versus RHE). EIS measurements were conducted at room temperature inside a homemade Faraday’s cage using a frequency range of 10^5^ to 0.01 Hz and voltage amplitude of 0.01 V_RMS_ AC amplitude, at 1.8 V vs RHE at 1 mol L^–1^ NaOH at pH 14. Electrocatalytic activity for OER was assessed by linear sweep voltammetry (LSV) from 0.5 to 2.0 V vs RHE under a scan rate of 10 mV s^–1^. Potentials were converted to the RHE scale by the following relation (eq 1)
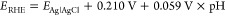
1

The overpotentials (η) for the OER were calculated according to eq 2.
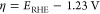
2

Tafel slope was determined by fitting the LSV results by eq 3
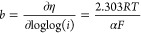
3where *b* is the Tafel slope in mV dec^–1^, η is the overpotential in V, *R* is the universal gas constant, *T* is the temperature in Kelvin, *F* is the Faraday constant, and α is the coefficient of anodic transfer.

The ECSA of the films were calculated from the electrochemical double layer capacitance (*C*_dl_) of the electrode, via CV scan, conducted in a non-Faradaic region at different scan rates of 20 to 100 mV s^–1^. The *C*_dl_ was obtained from the slope of the curve of the scan rate (V s^–1^) versus current density (mA). The specific formula was calculated as shown in eq 4
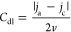
4where *v* is the scan rate for testing CV, *j*_a_ and *j*_c_ are the anodic and cathodic current densities at 0.53 V (vs RHE) fitted from the CV curve.

ECSA (cm^2^) was calculated by eq 5
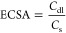
5where *C*_s_ is the specific capacitance (0.040) in alkaline solution in mF cm^–2^.^42^

The roughness factor (RF) values were calculated by eq 6 and *S*_geo_ is the geometric area of the electrode that is 1 cm^2^ for the FTO.
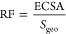
6

The specific activity (SA) (mA cm^–1^) was calculated by dividing the current *i* (mA) at η = 0.90 V by the ECSA. This calculation is expressed by eq 7
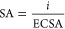
7

Faradaic efficiency (η_F_) was calculated using eq 8, where the volume of evolved oxygen was measured by volumetry, and the calculated theoretical volume was calculated from the Faraday equation.^43^
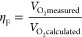
8

## 3 Results and Discussion

Aiming to enhance the OER electrocatalysis activity of low-cost and abundant elements, a trimetallic CoNiMn oxide film was synthesized by electrodeposition over FTO substrate using KCl as supporting electrolyte and activated afterward by CV in KOH. The overpotential at density current of 25 mA cm^–2^ was 430 mV for CoNiMn, against 800, 890, 600, 1000, 940, and 910 mV for CoMn, NiMn, CoNi, Mn, Ni, and Co, respectively, and shows the superior activity of the trimetallic film in comparison with the monometallic and bimetallic corresponding oxides, as will be discussed in detail later. Furthermore, an impressive 100 mV overpotential at 10 mA cm^–2^ was achieved by the CoNiMn film. To understand the synergistic effect between the first-row transition metal elements, a thorough characterization was carried out.

### 3.1 Structural and Morphological Characterization of the Films

**Figure 1:**
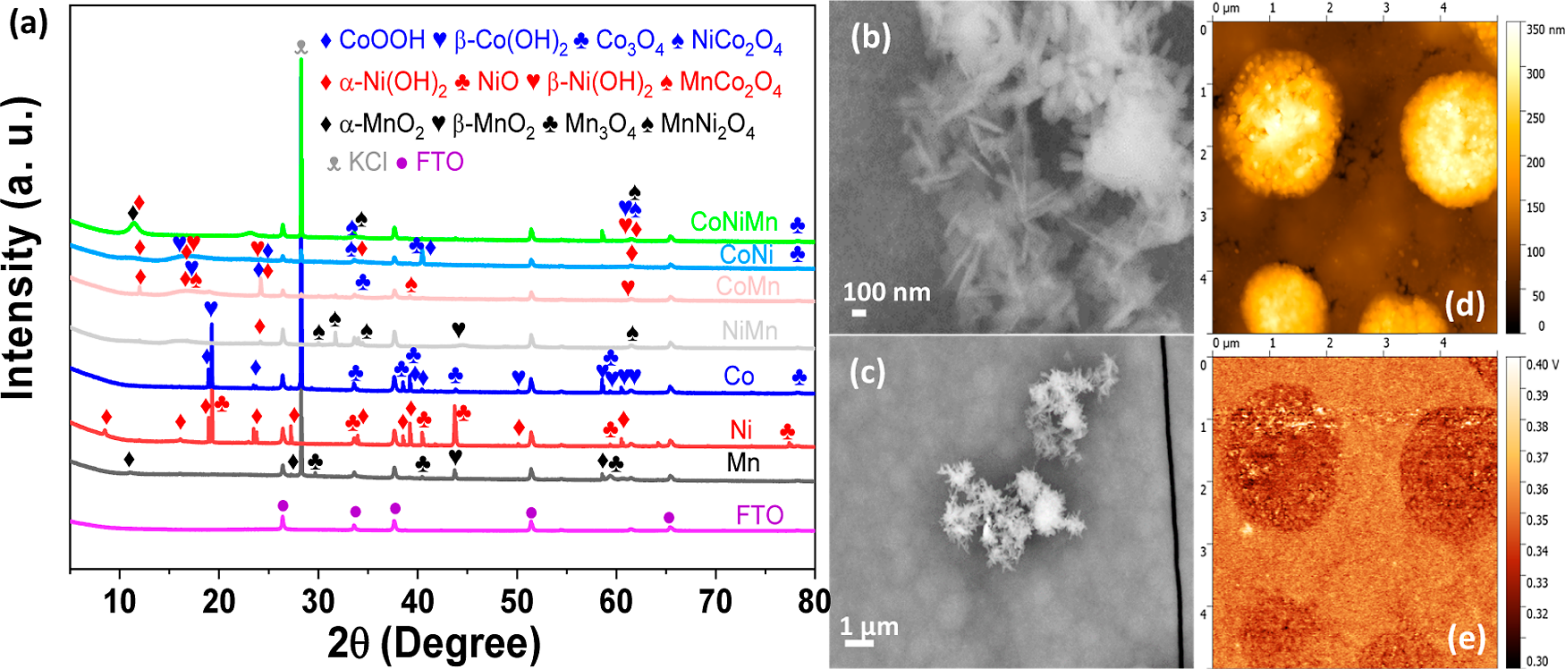
Powder XRD patterns of the films (a). SEM images (b,c), AFM (d), and KPFM (e) of the CoNiMn film.

The structural analysis of the films was carried out by the XRD technique. Figure 1a shows a typical XRD pattern depicting a mixture of phases observed in all films. This mixture comprises various oxides, including α-MnO_2_ (JCPDS 44-0141), β-MnO_2_ (JCPDS no. 024-0735), and Mn_3_O_4_ (JCPDS no. 24-0734) for the Mn film. The Ni film exhibited phases of α-Ni(OH)_2_ (JCPDS 38-0715), β-Ni(OH)_2_ (JCPDS 14-0117), and NiO (JCPDS 78-0423). The Co film exhibited phases of β-Co(OH)_2_ (JCPDS 30-0443), CoOOH (JCPDS 73-1213), and Co_3_O_4_ (JCPDS 42-1467). The bimetallic oxide NiMn presented the MnNi_2_O_4_ phase (JCPDS 36-0083), in addition to a mixture of phases observed in the Mn and Ni monometallic films. The bimetallic oxides CoMn and CoNi also exhibited new phases in addition to those found in the Mn and Ni monometallic films, such as the MnCo_2_O_4_ (JCPDS 32-0297) and NiCo_2_O_4_ (JCPDS no. 20-0781) phases, respectively. Additionally, the XRD analysis detected diffraction patterns of KCl (JCPDS 41-1476) and SnO_2_ (JCPDS 88-0287) in all films, with the latter associated with the FTO substrate. Lastly, the XRD pattern of the CoNiMn mixed film reveals that its composition consists of a combination of phases comprising the oxides derived from Co, Ni, and Mn single and bimetallic metals like α-MnO_2_, Ni(OH)_2_, Co(OH)_2_, MnNi_2_O_4_, MnCo_2_O_4_, and NiCo_2_O_4_, for example. Comparing with the elemental composition determined by ICP-OES (Table S1), the atomic ratio of the Co/Ni/Mn film is 4:1.6:1, revealing a predominance of cobalt in the film. The oxides were probably formed during the electrodeposition in KCl electrolyte and the hydroxides during the electrochemical activation in KOH.

**Figure 2:**
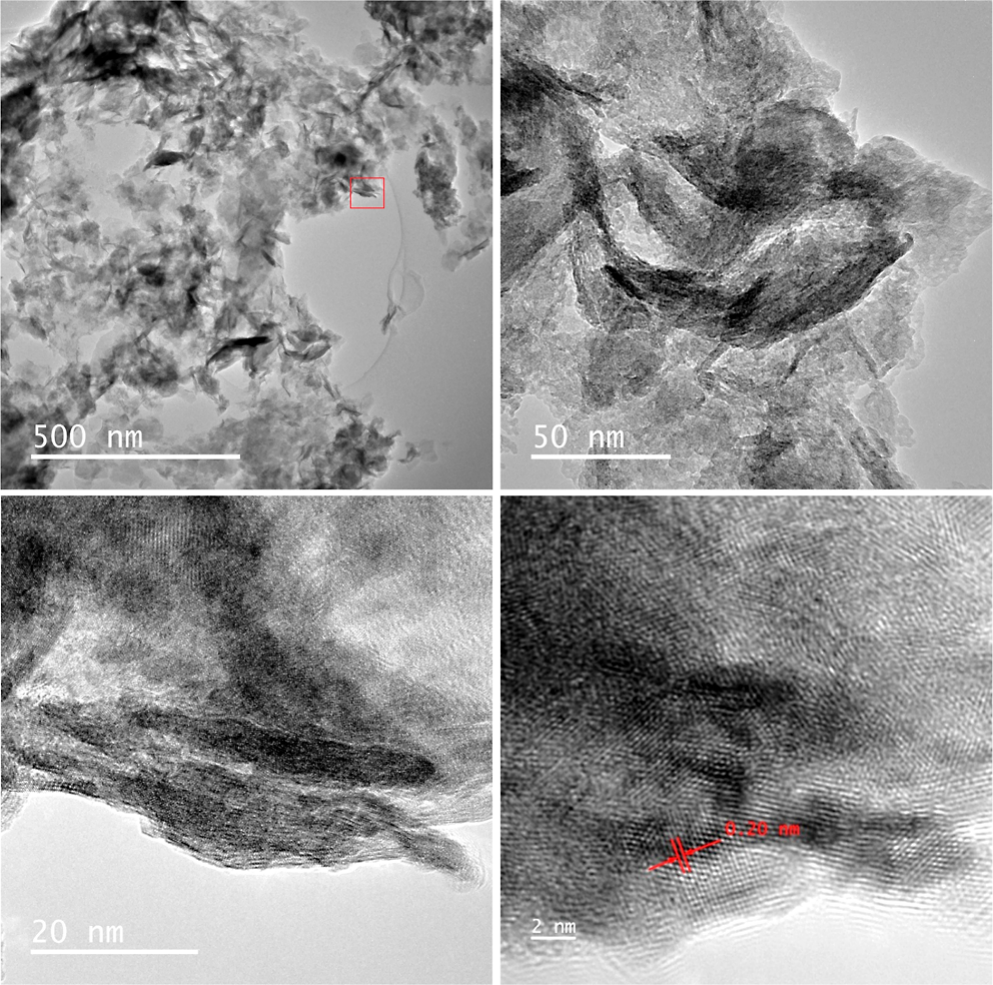
Low magnification (top) and high magnification (bottom) TEM images of the CoNiMn film.

SEM images of the electrodes indicate a uniform distribution of the CoNiMn film over the FTO substrate, as shown in Figures 1b,c and S3, exhibiting a well-defined morphology in the form of nanoneedles. Detailed morphological study by TEM revealed that the mixed film is composted mainly by nanoneedles, as observed in Figures 2 and S5. The nanoneedles are individually small, measuring less than 50 nm in length and much smaller in diameter, around 2 nm. However, when they aggregate, they create structures that bear a resemblance to nanosheets. A higher resolution image at the bottom of Figure 2 shows the crystallinity of the sample. The measured distance between the atom planes corresponds to the (400) plane of NiCo_2_O_4_. The energy dispersive spectroscopy (EDS-TEM mapping) of the CoNiMn film is shown in Figure S7 and confirmed that Co, Ni, Mn, and O are the main elements, and those are homogeneously distributed in the CoNiMn oxide. The atomic percentage of the elements is shown in Table S2, and the atomic ratio of Co/Ni/Mn is 6.5:1.7:1, similar to the bulk ratio determined by ICP-OES (4:1.6:1), but the higher Co amount in EDS suggests a prevalence of this element on the film surface.

**Figure 3:**
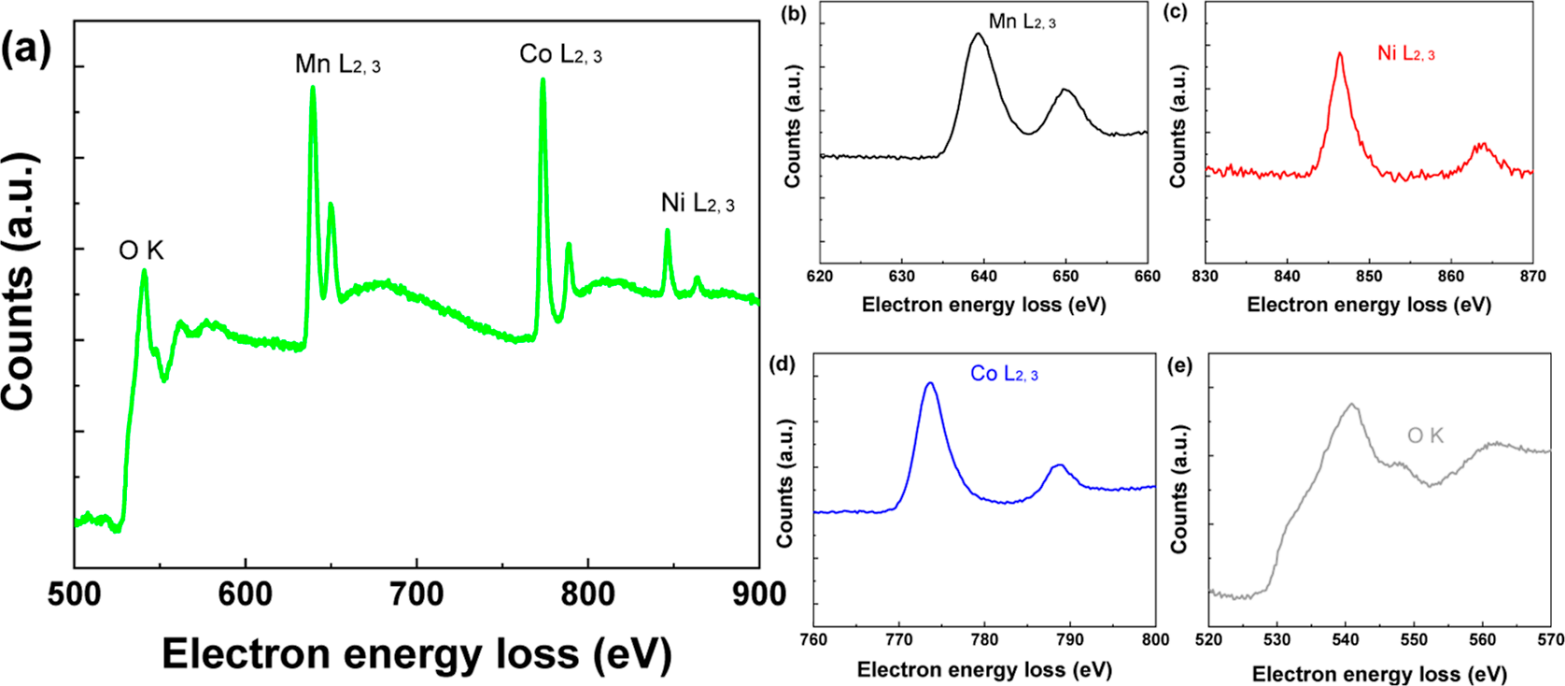
EELS of O, Mn, Co, and Ni for CoNiMn mixed oxide after background removal (a) and the isolated EELS region for Mn (b), Ni (c), Co (d), and O (e).

Figure 3 shows electron energy loss spectra (EELS) acquired during the TEM analysis. The background for each edge was subtracted by fitting a power-law model to the pre-edge and post-edge regions. The spectra are mainly constituted by the L_2_ and L_3_ edges of Mn at 649.88 and 639.30 eV, Co at 788.87 and 773.67 eV, and those of Ni at 864.11 and 846.54 eV, respectively. Since the L_2,3_ edges of Mn, Co, and Ni on the EELS spectrum are widely separated from each other as shown in Figure 3a–e, peak-overlap and consequent convolution issues are averted. Numerous researchers have observed that the relationship between both the L_3_/L_2_ height ratio and Δ*E* (L_2_–L_3_) with the oxidation states varies among different transition elements, but there is no universal rule.^44−53^Table 1 shows the L_3_/L_2_ height ratio and L_2_–L_3_ for the elements Mn, Co, and Ni in the CoNiMn film. As can be seen in Figure 3, both the energy loss near edge structure and extended electron energy loss fine structure of the oxygen K edge are different for all of the Mn, Co, and Ni species, indicating a mixed oxidation state of the three elements, as expected.

**Table 1:** L_3_/L_2_-Ratio and Difference *E*_L_3__ – *E*_L_2__ are Given for All Metal Oxides^a^

elements	L_3_/L_2_	*E*_O_ (eV)	*E*_L_3__ (eV)	*E*_L_2__ (eV)	Δ*E* (*E*_L2_ – *E*_L3_) (eV)
Mn	2.78	634.70	639.20	649.78	10.58
Ni	3.92	842.72	846.54	864.11	17.57
Co	3.39	769.36	773.67	788.87	15.20

a L_2,3_ edge onset *E*_O_, L_3_ peak maximum position, L_2_ peak maximum position, and *E*_L_3__,_L_2__ peak maximum position L_2_,L_3_.

In particular, to determine the oxidation state of cobalt, a comprehensive analysis of the L_2,3_ edge of cobalt and the K edge of oxygen in EELS spectra is highly efficient. The obtained spectra can be meticulously compared to existing EELS data in the literature, focusing on changes in energy and variations in edge shapes. A key indicator of the oxidation state of Co is the L_3_/L_2_ intensity ratio and the Δ*E*. A decrease in the L_3_/L_2_ ratio implies an increase in the oxidation state.^48,49^ According to the literature, the Δ*E* and L_3_/L_2_ for Co_3_O_4_ are 15.5 and 2.42 eV, respectively, while for CoO, they are 16 and 4.51 eV, respectively. A variation in the chemical shift of less than 1.5 eV can be observed for Δ*E*.^51^Figure 3d shows that the Δ*E* and L_3_/L_2_ for Co in CoNiMn sample is 15.2 and 3.39 eV, which can be assigned to the presence of Co^4+^, Co^3+^, and Co^2+^ species, consistent with the phases found in the XRD. The L_3_/L_2_ intensity ratio is particularly sensitive to oxidation state, with a higher ratio indicating a more oxidized state.^48,49^

The Mn EELS profile, as shown in Figure 3b, represents the L_3_ and L_2_ (L_2,3_) peaks, corresponding to the electronic excitations of the 2p_3/2_ and 2p_1/2_ core states for unoccupied 3d atomic orbitals, respectively. For Mn, the values of L_3_/L_2_ vary from 1 to 3 and that of Δ*E* vary from 10 to 11.5 eV. For CoNiMn, values for L_3_/L_2_ and Δ*E* were found to be 2.78 and 10.58 eV, respectively. The energy difference between these peaks, as well as for Co, varies within the range from Mn^3+^ to Mn^4+^.^56^ For example, Zhang et al.^54^ found that as the oxidation state increases, the L_3_/L_2_ ratio decreases for Mn. Tan et al.^53^ identified a strong correlation between the L_3_/L_2_ ratio and the integral width for Mn^2+^, but observed only marginal dependence for Mn^3+^ and Mn^4+^. The Ni peak at 846 eV may be related to the chemical bond between nickel and oxygen at the CoNiMn interface, and the Ni^2+^ state was observed.^55^ In contrast, the L_3_/L_2_ ratio for the Ni-_L_2,3__ edges in the Ni^2+^ state is lower than in the metallic state (Ni^0^), as observed by Bawane et al.^55^ Ni^3+^ presents a double peak in L_3_, often broadening the L_3_ peak, which was not observed.^47^

AFM measurements were also carried out to characterize the surface of the prepared films. With these measurements, it was possible to access the mean representativeness of the surface roughness of all samples (Figures 1d,e and S2), where clusters of nanoparticles were observed in the order of 2 μm and a maximum height of 500 nm with particle sizes in the nanometric order, close to 2 nm, corroborating the TEM results. Kelvin probe force microscopy measurements (KPFM) were also performed as a complementary technique for mapping the work function through areas of different potentials on the films. Distinct potential-contrast regions were observed in the analyzed film, with potential differences ranging from 0.25 to 0.48 V. Dark areas, characterized by an electronegative potential, extracted electrons from adjacent light areas. The film was found to consist of nanometer structures that formed agglomerates in certain regions.

### 3.2 Electrochemical Characterizations and OER Performance of the Films

**Figure 4:**
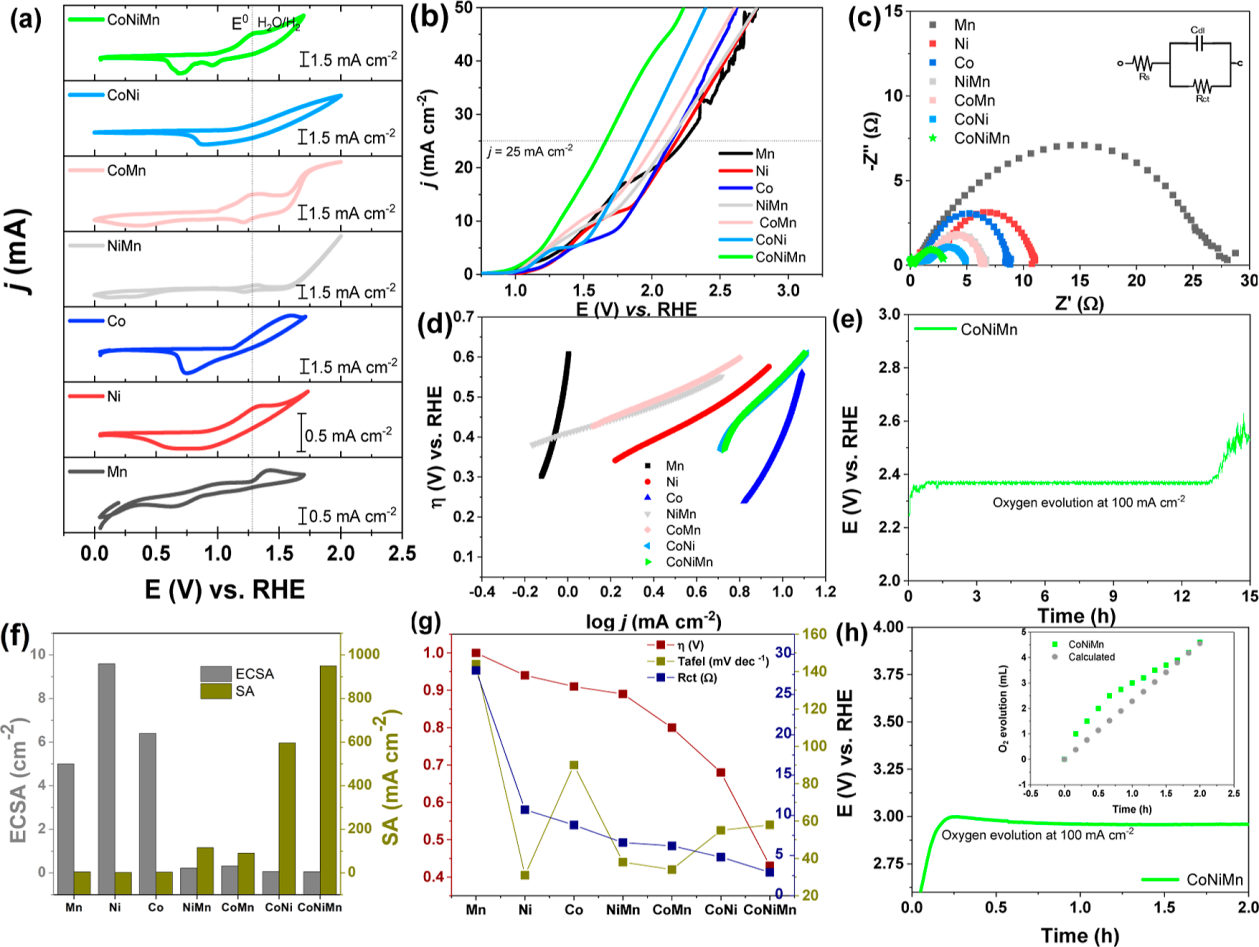
Electrochemical characterization of CoNiMn film: (a) voltammetric profile in 1 mol L^–1^ KOH, pH 14 before OER, (b) LSV curves, (c) Nyquist diagrams—inset the equivalent circuit, (d) Tafel curves, and (e) stability test with a constant current density of 100 mA cm^–2^ at 1 mol L^–1^ KOH on CoNiMn/FTO. (f) Comparison of the ECSA with SA; (g) comparison between the overpotential (η), Tafel, and *R*_ct_; and (h) chronoamperometry at a constant current density of 100 mA cm^–2^ at 1 mol L^–1^ KOH for 2 h—inlet Faradaic efficiency measured vs predicted by Faraday equation after holding the electrode at 100 mA cm^–2^ for 2 h.

Electrocatalytic activities of the electrodeposited CoNiMn/FTO film toward the OER were first evaluated by polarization curves (LSV curves). Next, the electrochemical performances were evaluated by parameters like electrochemical double-layer capacitance, charge transfer resistance, electrochemical active surface area, SA, Tafel slope, overpotential values, and Faradaic efficiency from data acquired by CV, EIS, LSV curves, and chronopotentiometry. The low overpotentials, small Tafel slopes, and low charge transfer resistance achieved by the CoNiMn mixed oxide film demonstrated their outstanding catalytic activities for OER, as shown in Figure 4. The superior performance of the CoNiMn film in relation to the monometallic and bimetallic films is evidence of the synergistic effect between the elements. The multiple oxidation states of the elements can facilitate electron transfer between them and stabilize the catalytic intermediates of high valence that are key to perform the OER.

CV was performed to investigate the oxidation/reduction processes of the films after the activation, as it is known that there are changes in the crystalline structure of the oxides as a function of potential variation during CV.^56^ All of the potentials in this study were converted to the RHE scale. Whenever works that do not use the RHE scale are referenced, the scale will be explicitly mentioned. CV measurements conducted in a 1 mol L^–1^ in KOH solution at pH 14 with a scan rate of 50 mV s^–1^ are shown in Figure 4a. The different metal oxide films displayed distinct profiles concerning both the number of oxidation and reduction peaks as well as the current density. The current density escalated toward the mixed oxide, with Ni exhibiting the lowest and Co the highest values among the monometallics, and NiMn exhibiting the lowest current densities among the bimetallics, in the order Ni < Mn < Co < NiMn < CoMn < CoNi < CoNiMn. The oxide surfaces are dynamic, altering the oxidation state of the metals as the CV potential varies. In previous studies, within the same potential range as in this investigation, the transition in the oxidation state of the metal species changes from M(II) ↔ M(III) and M(III) ↔ M(IV), respectively.^57−60^ The latter oxidation state is less common for metals, such as Ni and Co. The oxidation peaks for Mn were identified at 0.98 and 1.40 V, with corresponding reduction peaks at 1.14 and 0.66 V. Conversely, for Ni and Co, only one oxidation peak was observed at 1.33 and 1.58 V, respectively. Regarding the reduction peak, Co exhibited a well-defined peak at 0.74 V, while Ni showed a broadened peak spanning from 1.0 to 0.5 V, possibly due to capacitive effects, akin to observations made by Aguilera et al.,^61,62^ where such effects were linked to ion diffusion challenges within the material. The bimetallic oxides displayed distinct characteristics. The NiMn oxide exhibited two oxidation peaks at 0.63 and 1.28 V and two reduction peaks at 1.21 and 0.32 V, respectively. In contrast, the CoMn and CoNi mixed oxides each exhibited a single oxidation peak at 1.29 and 1.30 V, respectively. However, while the CoMn mixed oxide showed a single large reduction peak at 0.93 V, the CoNi oxide displayed two reduction peaks at 1.22 and 0.86 V, respectively. In the case of the trimetallic mixed oxide, a single oxidation peak appeared at 1.28 V, accompanied by three reduction peaks at 0.95, 0.82, and 0.68 V, respectively. Notably, the reduction peak potentials of the CoNiMn film differed slightly from those of the individual metals; however, the current densities were higher, indicating enhanced electrochemical properties of the mixed material compared to the pure oxides.

The electrolyte resistance (*R*_s_) and charge transfer resistance (*R*_ct_) were analyzed by EIS measurements, and the equivalent circuit (Figure 4c-inlet) was fitted for the Nyquist plots depicted in Figure 4c. EIS was performed at 1.8 V vs RHE between 0.1 Hz and 0.1 MHz before the OER tests. The *R*_s_ and *R*_ct_ are associated with the kinetics of the charge transfer processes at the solution and at the electrode/film interface, respectively, while *C*_dl_ represents the electron transfer at the electrode/electrolyte interface and all of them are present in all aqueous systems. In addition, the *R*_ct_ is related to the combined steps of the OER, i.e., assigned with the overall rate of OER. The *R*_ct_ values of 28.0, 10.8, 8.4, 7.9, 7.4, 4.9, and 3.0 Ω were found for the Mn, Ni, Co, NiMn, CoMn, CoNi, and CoNiMn films (Table 2), respectively, showing the synergism between the elements in improving the conductive property of the trimetallic film and increasing the efficiency of the OER.

**Table 2:** OER Electrocatalytic Data^a^

film	*R*_ct_ (Ω)	*j*_,900_ (mA cm^–2^)	ECSA (cm^2^)	η_10_ (mV)	η_25_ (mV)	SA_900_ (mA cm^–2^)	Tafel slope (mV dec^–1^)
Mn	28.0	21.8	5.0	380	1000	4.4	144
Ni	10.8	22.8	9.6	330	940	2.4	31
Co	8.4	24.2	6.4	370	910	3.8	90
NiMn	7.9	25.5	0.22	325	890	115.9	38
CoMn	7.4	28.9	0.32	280	800	90.3	34
CoNi	4.9	35.8	0.060	328	680	596.7	55
CoNiMn	3.0	45.6	0.048	100	430	950.0	58

a Overpotential (η_10_) at *j* = 10 mA cm^–2^ (η_25_) at *j* = 25 mA cm^–2^ and SA at η = 900 mV.

The LSV curves of the films were investigated to estimate the electrocatalytic OER performance carried out in 1 mol L^–1^ KOH, as shown in Figure 4b. Table 2 presents the respective overpotentials measured at 10 and 25 mA cm^–2^. To drive a current density of 25 mA cm^–2^, the Mn, Ni, Co, NiMn, CoMn, CoNi, and CoNiMn samples require an overpotential of 1000, 940, 910, 800, 890, 680, and 430 mV, respectively. These overpotentials are considerably small due to the electrochemical activation, but the trimetallic mixed oxide film stands out with an extremely low overpotential, comparable to or better than those of other state-of-the-art trimetallic oxides’ OER electrocatalysts at alkaline electrolyte (pH 14) listed in Table S3. Furthermore, impressive overpotential of 100 mV at 10 mA cm^–2^ was reached by CoNiMn, showing its outstanding electrocatalytic activity toward OER. Nanostructured ball-milled Ni–Co–Mn oxides synthesized from spent Li-ion batteries showed an overpotential of 367 mV at 10 mA cm^–2^.^63^ Nanocast mixed Ni–Co–Mn oxides with controlled surface and pore structure of the type Ni_*x*_Co_*y*_Mn_*z*_O_4_ calcined at different temperatures achieved overpotentials at 10 mA cm^–2^ of 429 mV (300 °C), 436 mV (500 °C), and 465 mV (700 °C), which after activation by 200 cycles of CV improved the OER activity, reaching overpotentials of 402, 415, and 458 mV at the respective temperatures.^21^ The CoNiMn films synthesized in this work present superior OER activity and need only one CV cycle to reach very low overpotentials.

The Tafel slope values of Mn, Ni, Co, NiMn, CoMn, CoNi, and CoNiMn (Figure 4d) were 144, 31, 90, 38, 34, 55, and 58 mV dec^–1^, respectively. The smallest Tafel slope of CoNiMn indicates a faster reaction kinetics, i.e., prominent electrocatalytic OER performance. As shown in Figure 4g, there is a correlation between low overpotential, low Tafel slope, and low *R*_ct_ of the CoMnNi/FTO film that demonstrated its outstanding catalytic activity for the OER in comparison to the single and bimetallic metal oxides due to a synergistic effect between the elements that improved the electronic and kinetic OER properties.

Another important parameter to evaluate the efficiency of the electrocatalyst is the ESCA, which is estimated by measuring the electrochemical double layer capacitance acquired through CV measurements in non-Faradaic region (Figures S10 and S11). Higher ECSA values mean more electrochemically active sites exposed to the electrolyte, which is favorable for oxygen gas generation during OER. The ECSA values shown in Table 2 followed the order Ni > Co > Mn > CoMn > NiMn > CoNi > CoNiMn. The monometallic films presented higher ECSA than the bimetallic ones, which presented higher ECSA than the trimetallic films.

In order to compare the intrinsic electrocatalytic activities of the films regardless of the ECSA, the SA was calculated by dividing the current at η = 900 mV by the ECSA (eq 7), and the SA followed the order: CoNiMn > CoNi > NiMn > CoMn > Mn > Co > Ni (Figure 4f and Table 2). The trimetallic CoNiMn film presented the higher SA, and although it also presented the lower ECSA, its catalytic sites are more active than the bimetallic and monometallic. The SA of CoNiMn is a combination of the inherent activity of the metals Co, Mn, and Ni, and the catalytic sites of Co give the higher contribution for the CoNiMn overall performance. A similar trend is observed for the bimetallic oxides in comparison with the monometallic ones. This can also be discussed in terms of RF that is higher for the monometallic films, rendering it more porosity. While porous films have higher ECSA, this can hinder the mass transport of OH^–^ into and O_2_ out of the porous electrodes, which can cause internal active sites within the porous film to become catalytically inaccessible during OER operation. The improved OER activity observed for CoNiMn can also be attributed to the adjustable OH^–^ adsorption and O_2_ desorption capabilities arising from the contribution from the mixture of oxides, in contrast to the less active pure oxides in the OER. A decrease in accessibility to internal active sites by the high RF would result in a lower SA for the films containing Mn compared to the CoNiMn film.^42^ The analysis of SA should only be employed as a rough guide for comparing activity rather than an absolute indicator for efficient OER activity. This is particularly important when comparing high surface area and nanoporous films. The formation of CoNiMn by combining these three single-atom oxides resulted in a remarkable enhancement in the OER activity because of the synergistic effect between the elements.

The stability of the CoNiMn film (Figure 4e) was evaluated through chronoamperometry at a higher current density of 100 mA cm^–2^ over 15 h, with the CoNiMn film prepared with a deposition time of 100 s. Notably, the CoNiMn film exhibited an initial increase in the overpotential after 13 h. The Faradaic efficiency (Figure 4h) was followed by a stable performance during 2 h. In parallel, the Faradaic efficiency of the CoNiMn catalyst was determined by the measurement of the oxygen evolved over time by volumetry (Figure 4h-inlet), resulting in 100% of η_F_.

SEM (Figure S4), HRTEM (Figure S6), TEM–EDS (Figure S7), TEM-EELS (Figure S9 and Table S3), and CV analyses were conducted after long-term electrocatalytic stability test by chronopotentiometry at 100 mA cm^–2^ for 15 h for the CoNiMn/FTO film and no significant morphological and structural changes occurred on the catalyst, although an appearance of crystals identified by EDS as predominantly K and O was observed, attributed to exposure to KOH for 15 h. This shows the high stability of the CoNiMn films under harsh OER conditions.

## 4 Conclusions

The CoNiMn mixed oxide film was synthesized by electrodeposition and electrochemical activation by CV, forming a mixture of Co, Ni, and Mn oxides and hydroxides as the main phases. The nano-oxides were composed of different oxidation states M(IV), M(III), and M(II) originating from Mn, Ni, and Co, which could facilitate electron transfer between the elements and favor high-valent metal-oxygenated intermediates that are key for the OER catalytic cycle. The CoNiMn exhibited outstanding catalytic activity for the OER with low overpotentials of 100 and 430 mV at 10 and 25 mA cm^–2^, respectively, better or comparable to the state-of-art trimetallic oxides reported so far. Small Tafel slopes, low charge transfer resistance, and high SA, in comparison with the monometallic and bimetallic oxides, are also attributed to the superior performance of the trimetallic film. The CoNiMn film was stable up to 13 h at 100 mA cm^–2^, corresponding to a Faradaic efficiency of 100% during chronoamperometry. Furthermore, no structural or morphological change in the film CoNiMn after chronoamperometry was observed. In conclusion, the superior electrochemical properties and enhanced OER activity of the CoNiMn catalyst may stem from the synergistic effect between the metal elements, which resulted in a promising electrocatalyst for the OER based on low-cost and abundant elements.

## Supporting Information Available

Additional characterization data of the films: ICP-OES, optical microscopy, AFM, SEM, TEM, TEM–EDS, EELS, CV, *C*_dl_, and Tafel plots (PDF)
