# Supplementary material for: Electrocatalysis of Oxygen Evolution Reaction Promoted by CoNiMn Films Synthesized by Electrodeposition
Source: ACS Omega. 2024 Oct 17;9(43):43503–12. doi: 10.1021/acsomega.4c05057 (PMC11525539; doi:10.1021/acsomega.4c05057)
Supplement: Supplementary file 1 — ao4c05057_si_001.pdf [file ao4c05057_si_001.pdf]

Electrocatalysis of Oxygen Evolution Reaction Promoted by CoNiMn Films Synthesized by Electrodeposition

Ana Luisa Silva[1,*], Marcos V. Colaço[2], Liying Liu[3], Yutao Xing[4], Nakédia M. F. Carvalho[1,*]

[1] Universidade do Estado do Rio de Janeiro (UERJ), Instituto de Química, Rua São Francisco Xavier, 524, 20550-900 - Rio de Janeiro, RJ, Brasil.

[2] Universidade do Estado do Rio de Janeiro (UERJ), Instituto de Física, Rua São Francisco Xavier, 524, 20550-013 Rio de Janeiro, RJ, Brasil.

[3] Centro Brasileiro de Pesquisas Físicas (CBPF), Rua Doutor Xavier Sigaud 150, 22290180 - Rio de Janeiro, RJ – Brasil.

[4] Universidade Federal Fluminense, Instituto de Física, Niterói, RJ, Brasil, 24210–346.

* Corresponding author: Nakédia M. F. Carvalho – e-mail: nakedia@uerj.com; Ana Luisa Silva – e-mail: analuisa.als89@gmail.com

E-mail of co-authors: mvcolaco@gmail.com (Marcos Vinicius Colaço Gonçalves); xy@id.uff.br (Yutao Xing); lyliu.xing@gmail.com (Liying Liu)

Summary

S.1. Electrocatalysts characterization…………………………………….……………S3

S.1.1. Inductively coupled plasma optical emission spectrometry……….........……S3

S.1.2. Optical microscopy…….……………………………………………………..S4

S.1.3. Atomic force microscopy…………………………………….......................S5

S.1.4. Scanning electronic microscopy……………………………….......................S6

S.1.5. Transmission electronic microscopy …………..……………….......................S7

S.1.5. Cyclic voltammetry…………………………………………………..….....S12

S.1. Electrocatalysts characterization

S.1.1 Inductively coupled plasma optical emission spectrometry

The digestion of the films took place within the PTFE vessels, where an FTO substrate was introduced along with the corresponding electrodeposited film. The digestion solution comprised 5 mL of 20 mol L^{-1} HNO_3. After 10 minutes, no film residue was evident on the FTO surface, so the FTO was removed from the PTFE vessels. Subsequently, deionized water was introduced into the digested material within the PTFE vessels until reaching a total volume of 25 mL.

Table S1: The limits of detection and quantification, expressed in mg L^{-1}, were determined for electrodeposited films through digestion with HNO_3 for the analysis of Co, Mn, Ni, and Sn using ICP-OES.

Sample	Co (mg L^{-1})	Mn (mg L^{-1})	Ni (mg L^{-1})	Sn (mg L^{-1})
CoNiMn	17,9 ± 0,1	4,2 ± 0,1	7,2 ± 0,1	< LOD
LOD =	0,01	0,01	0,01	0,09
LOQ =	0,03	0,03	0,03	0,28

*The results found in the samples represent the average of three determinations with a confidence level of 95%.

The limits of detection (LOD) and quantification (LOQ) were calculated using Equations (1), (2), respectively.

LOD = 3.29* *sd/s* (1)

LOQ = 10 * *sd/s* (2)

where "*sd*" is the standard deviation of ten measurements of the blank solution (for each sample preparation procedure) and "*s*" is the slope of the analytical curve. All values were corrected for sample mass and dilutions require.

S.1.2 Optical microscopy

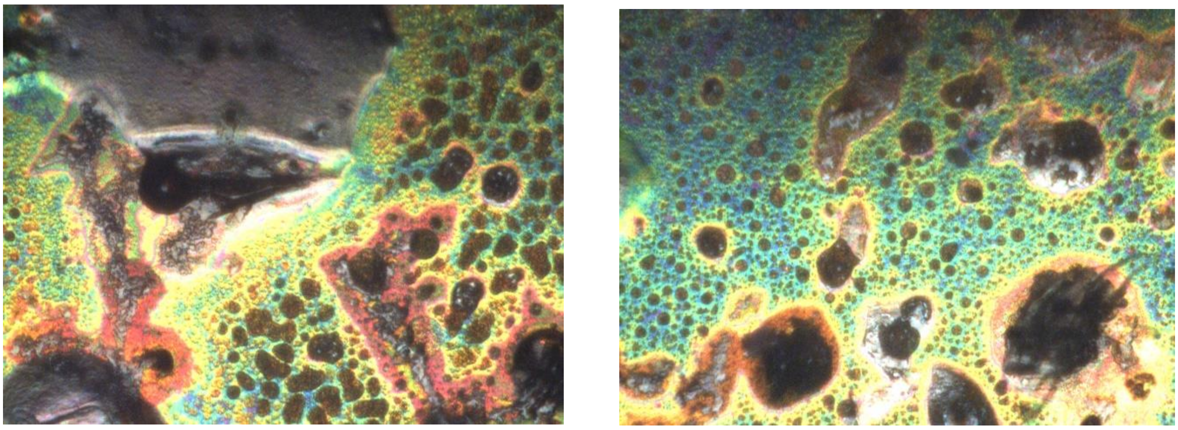

Fig S1. Optical images extracted from the optical microscope coupled to the atomic force microscope of the CoNiMn.

S.1.3 Atomic force microscopy

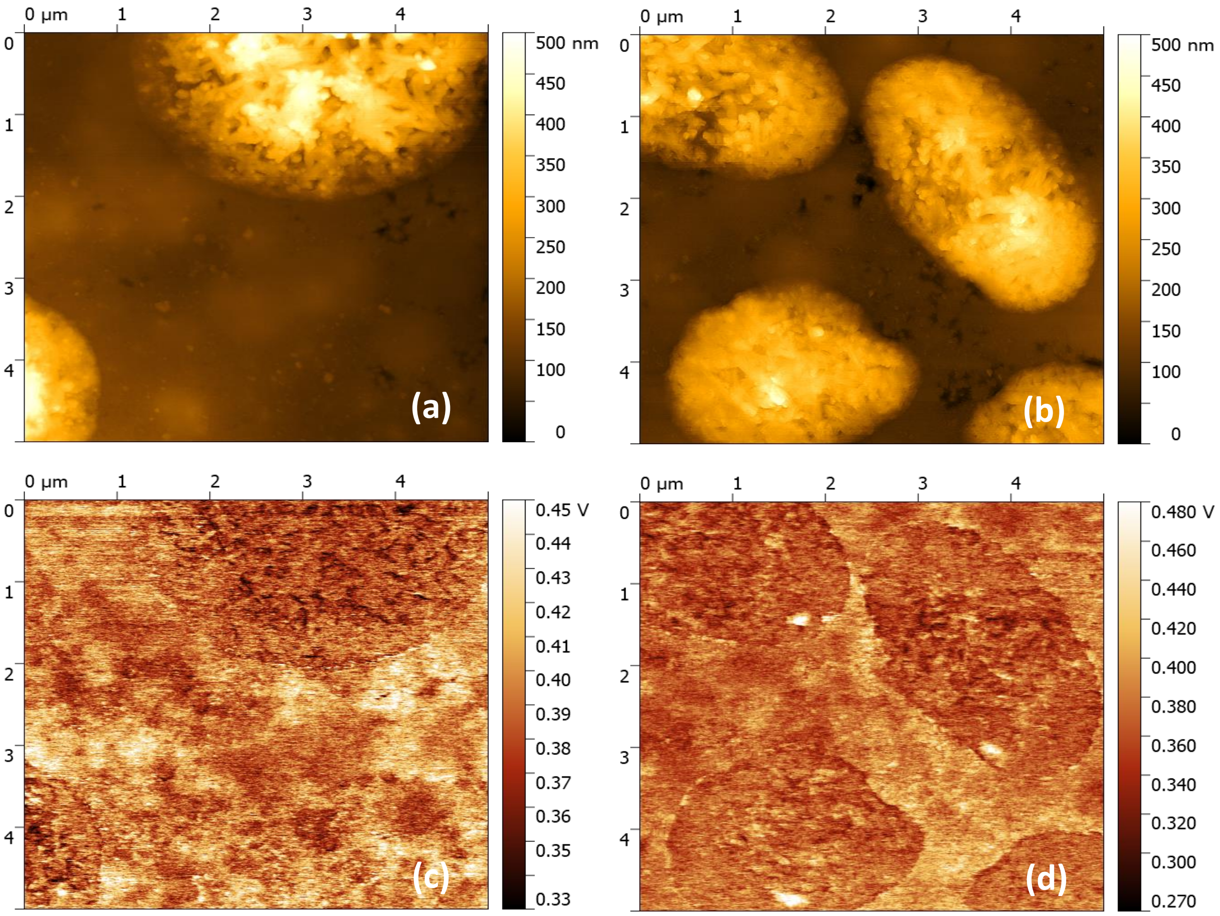

Fig S2. AFM images (A-B) and KPFM images (C-D) of the CoNiMn.

S.1.4 Scanning electron microscopy

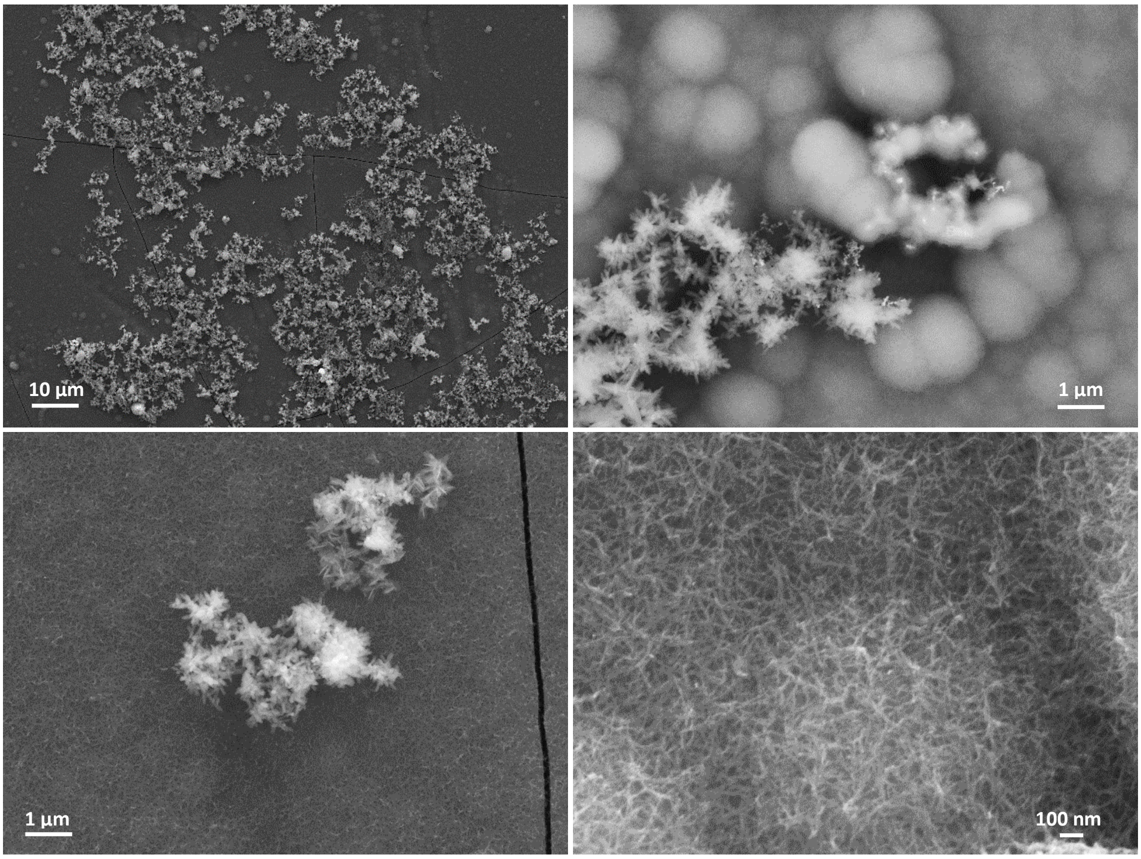

Fig S3. SEM image of the CoNiMn before OER.

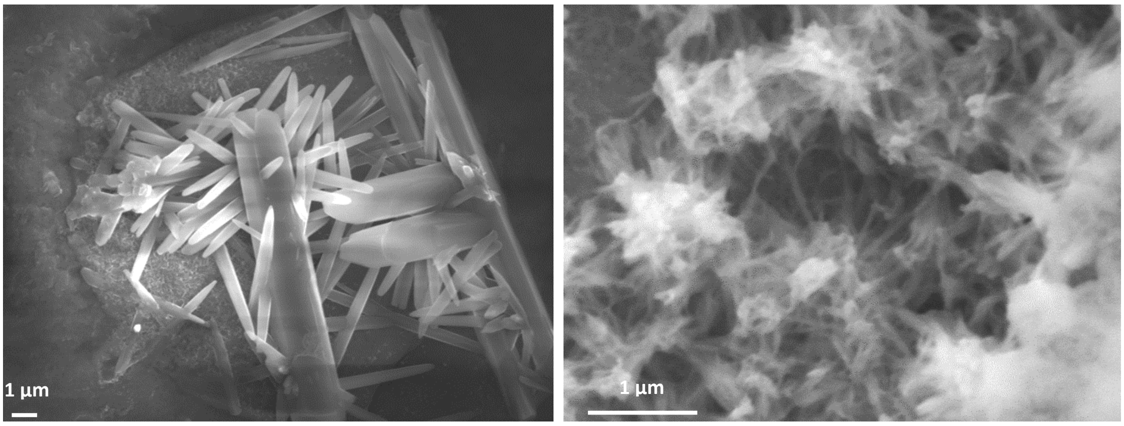

Fig S4. SEM image of the CoNiMn after OER.

S.1.5 Transmission electron microscopy

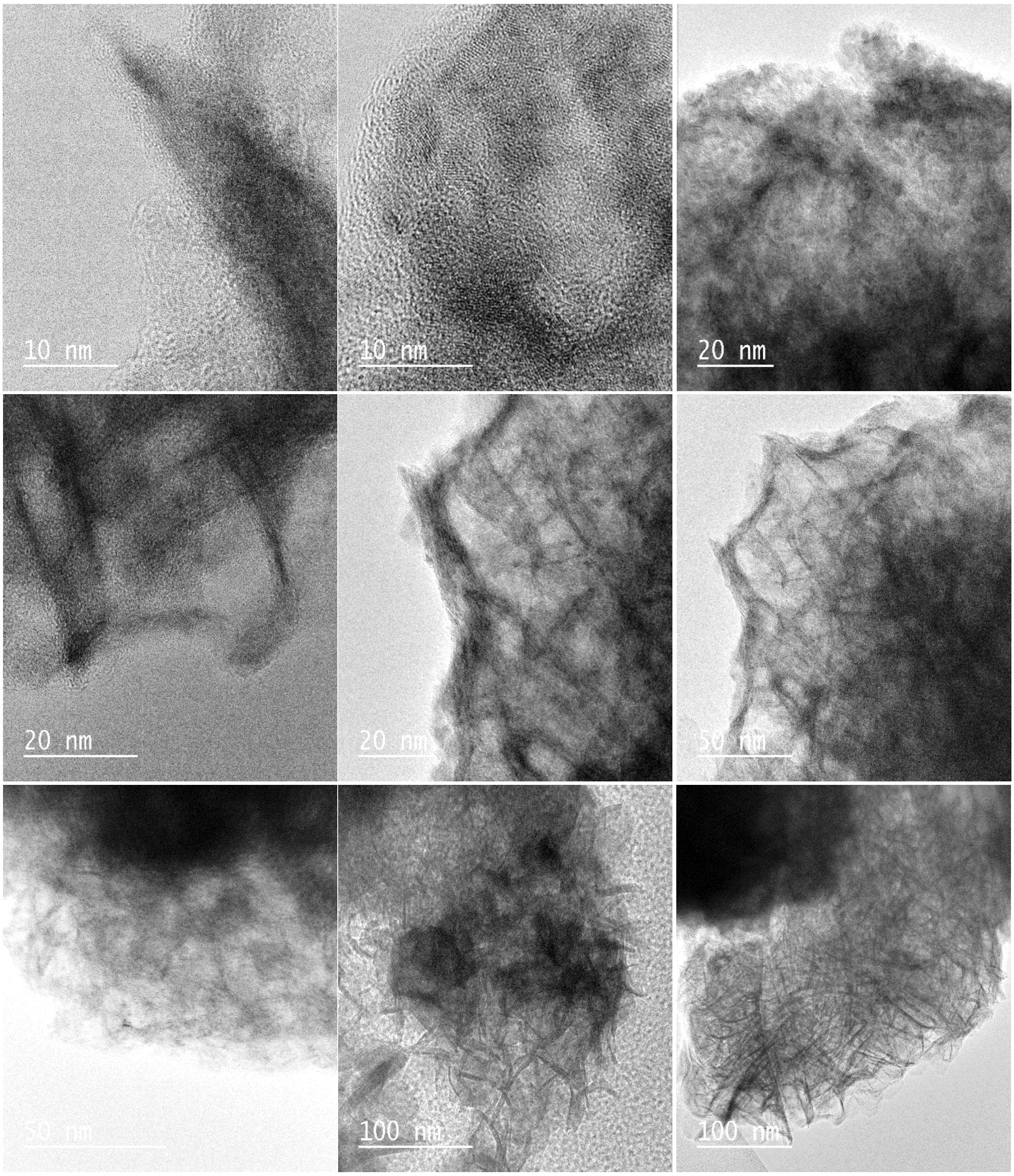

Fig S5. Low magnification and high magnification TEM imagens of the CoNiMn before OER.

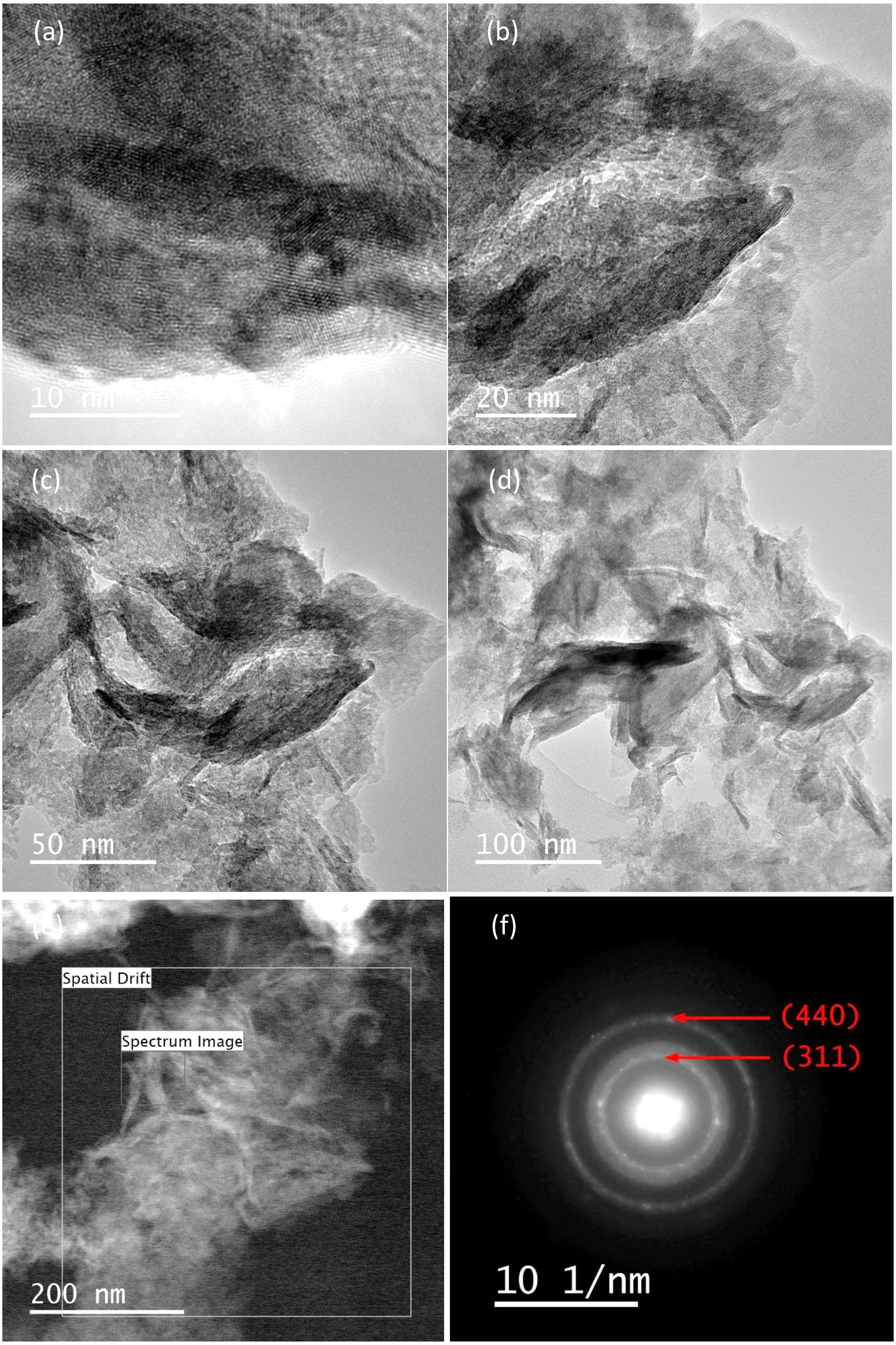

Fig S6. Low magnification and high magnification TEM imagens of the CoNiMn after OER (a-e) and (f) SAED pattern of $CoNi(OH)_2$.

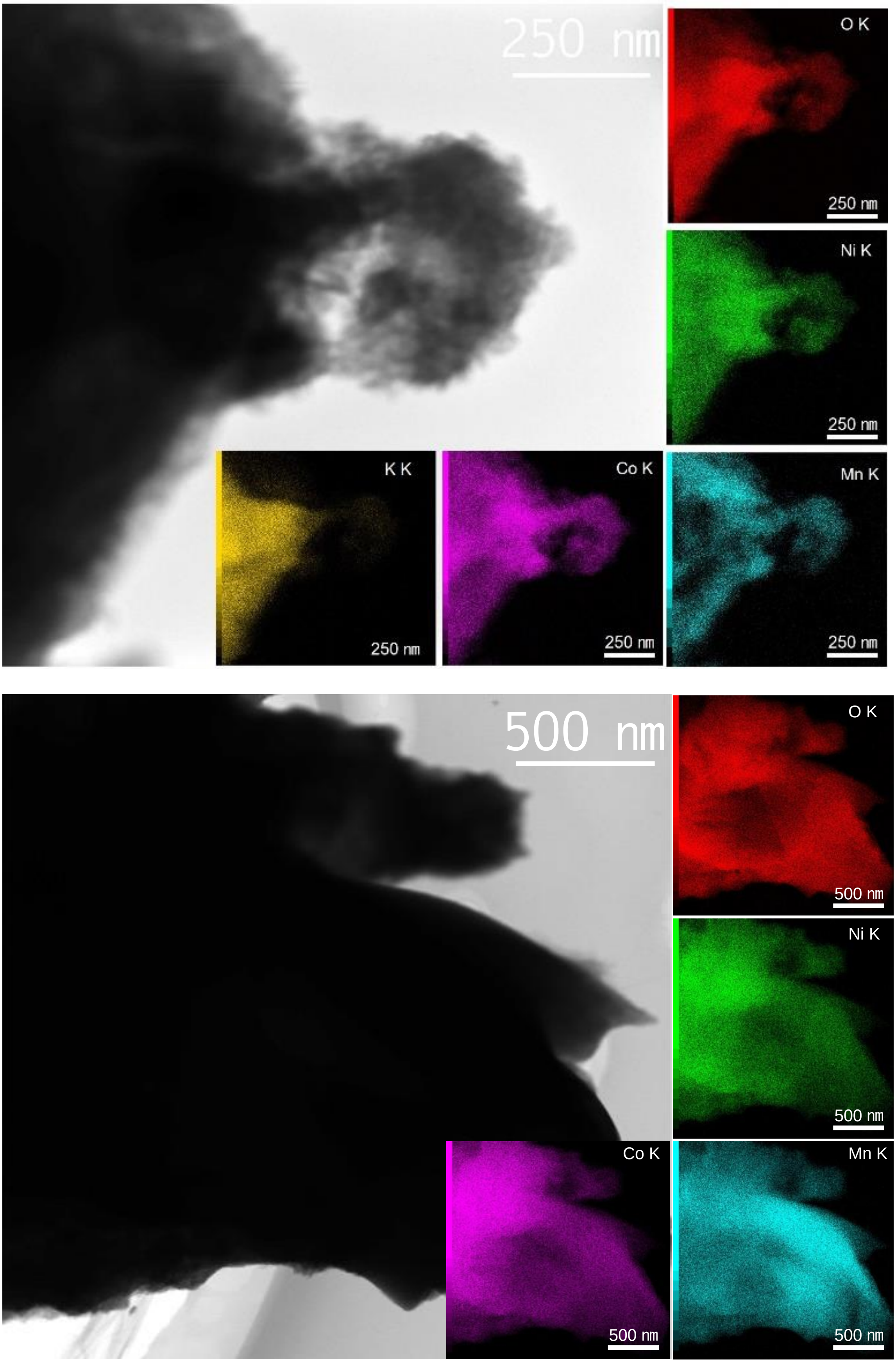

Fig S7. TEM-EDS mapping of the CoNiMn sample before (top) and after (bottom) of OER.

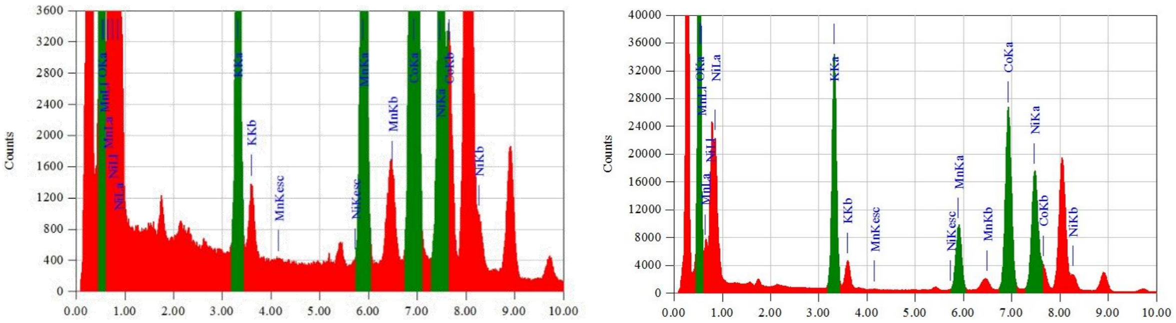

Fig S8. EDS survey of the CoNiMn sample.

Table S2. Average atomic percentage* of all catalysts

CoNiMn				
**O / at%**	**K / at%**	**Mn / at%**	**Ni % at**	**Co % at**
29.82	9.72	6.61	11.06	42.79

*Measured from EDS spectra

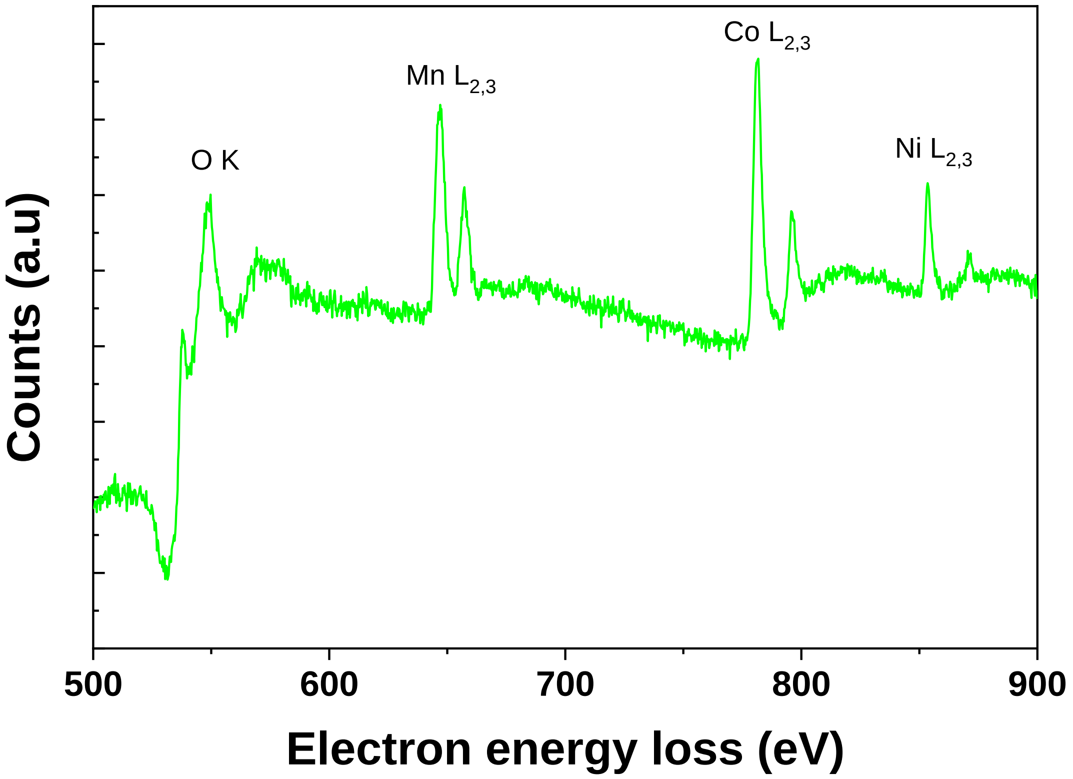

Fig S9. Electron energy loss spectra of O, Mn, Co and Ni for CoNiMn mixed oxide after OER.

Table S3. L_3/L_2-ratio and difference E_{L3}−E_{L2} are given for all metal oxides after OER.

**Elements**	**L_3/L_2**	**E_O (eV)**	**E_{L3} (eV)**	**E_{L2} (eV)**	**ΔE (E_{L2}−E_{L3}) (eV)**
Mn	1.89	641.88	646.65	657.33	10.68
Ni	3.92	849,71	853.40	870.58	17.18
Co	3.21	775.85	781.83	795.22	13.39

* $L_{2,3}$ edge onset E_O, L_3 peak maximum position, L_2 peak maximum position and $E_{L3,L2}$ peak maximum position L2,L3.

The results show that there was no change in the appearance of the peaks—such as broadening, narrowing, or the development of shoulders—indicating that the structure of the catalyst remained unchanged. Although there were shifts in the position of the peaks, this drift is common, particularly when measurements being compared are not taken on the same day.

S.1.6 Cyclic voltammetry

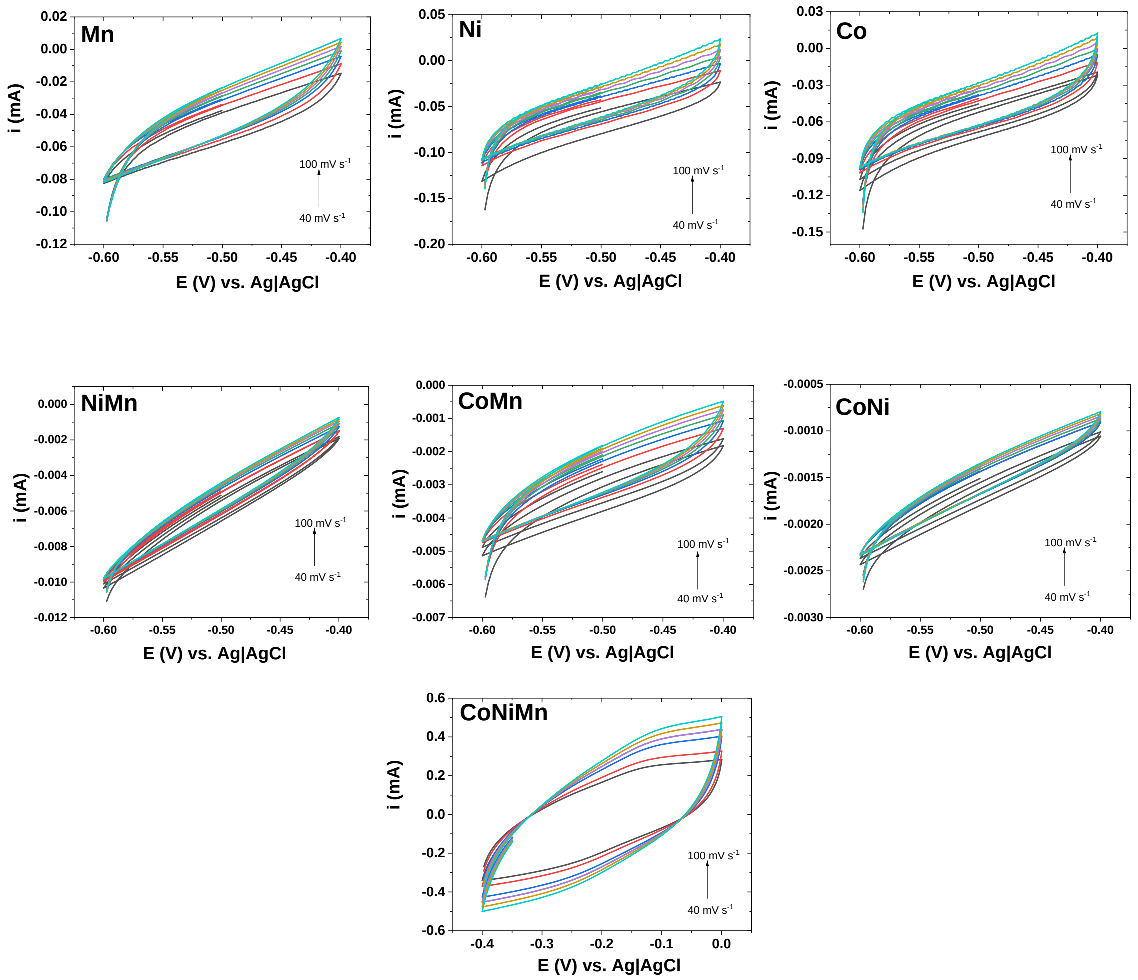

Fig S10. CVs in the non-faradaic region of the samples at different scan rates from 20 to 100 mV s^{-1}.

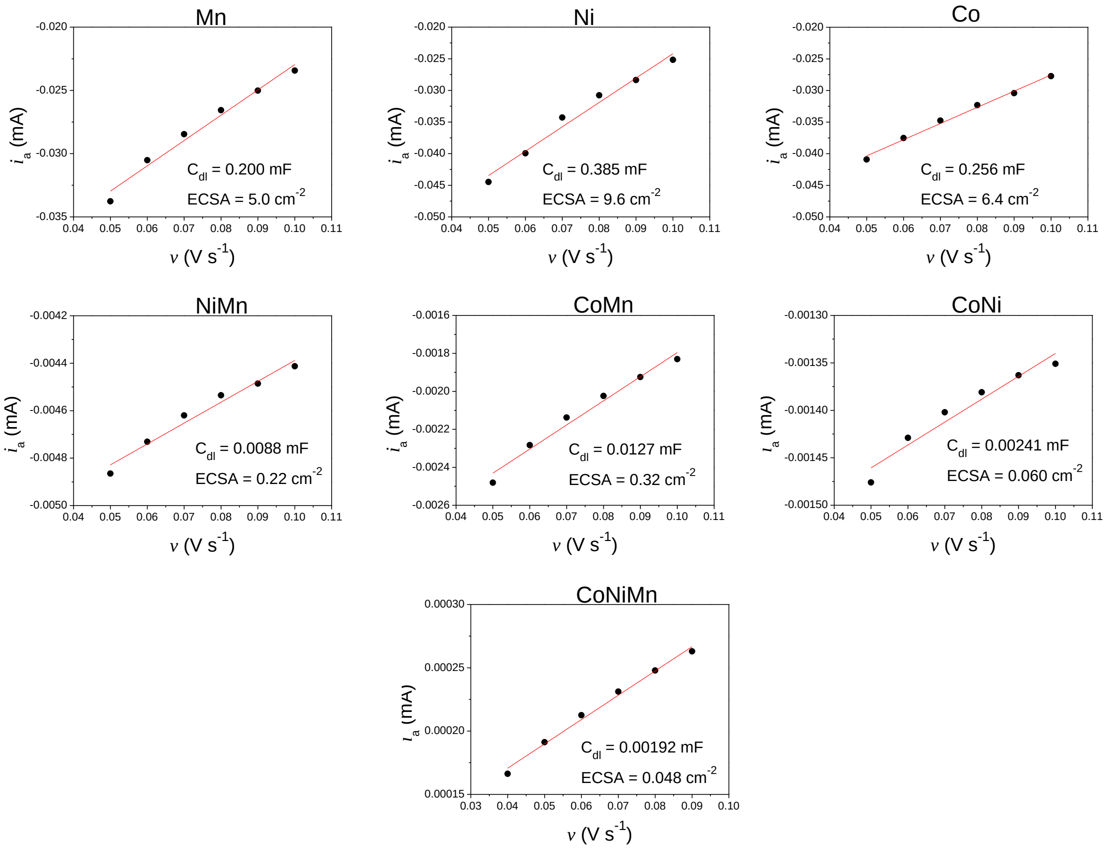

Fig S11. Values of the electrochemical double layer capacitance (Cdl) calculated from CVs data in Fig. S10.

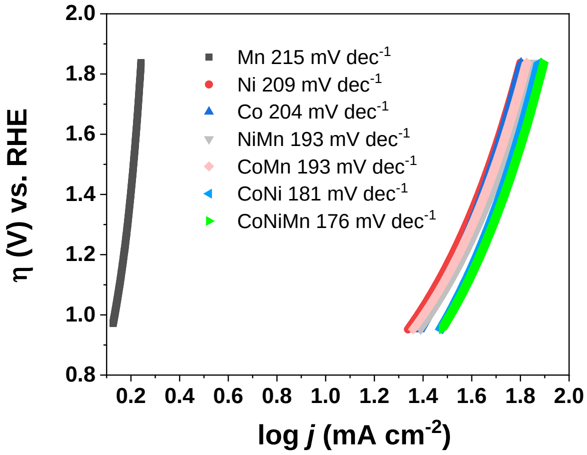

Fig S12. Tafel slope values calculated at higher overpotentials from LSV curves in Fig. 4(b).

Table S4. Comparison of OER catalytic activity of CoMnNi/FTO with some representative transition metal-based OER electrocatalysts reported in 1.0 M KOH solution.

Catalyst	Current density (mA cm^{-2})	Overpotential (mV)	Tafel Slope (mV dec^{-1})	Refs.
**CoNiMn**	10	100	58	This work
**CoNiMn**	25	430	58	
**Co**	25	910	90	
**Ni**	25	940	31	
**Mn**	25	1000	114	
**CoNi**	25	680	55	
**CoMn**	25	800	38	
**NiMn**	25	890	34	
**$Ni_xCo_yMn_zO_4$-300**	10	429	74	1
**$Ni_xCo_yMn_zO_4$-300 activated**	10	402	85	1
**$Ni_xCo_yMn_zO_4$-500**	10	436	78	1
**$Ni_xCo_yMn_zO_4$-500 activated**	10	415	79	1
**$Ni_xCo_yMn_zO_4$-700**	10	465	84	1
**$Ni_xCo_yMn_zO_4$-700 activated**	10	458	97	1
**Ni-Co-Mn oxide**	10	367	43.84	2
**$Fe_{0.05}Co_{0.09}W_{0.61}O_{0.25}$**	10	267	34.9	3
**$Ni_{0.75}Mn_{0.25}$ Oxide**	10	600	60	4
**Mo-$_{0.003}$-Ni/NC**	10	141	75.2	5
**Mo-$_{0.002}$-Ni/NC**	10	188	87.6	5
**NiFeCr**	10	250	29	6
**Co_3O_4 nanorods**	10	606	200	7
**Co_3O_4 nanorods**	3.7	385	70	7
**Co_3O_4 nanocube**	10	400	60	8
**$(Co_{2.7}Mn_{0.3})O_4$**	10	490	35.8	9

References

(1) Priamushko, T.; Guillet-Nicolas, R.; Yu, M.; Doyle, M.; Weidenthaler, C.; Tuÿsüz, H.; Kleitz, F. Nanocast Mixed Ni-Co-Mn Oxides with Controlled Surface and Pore Structure for Electrochemical Oxygen Evolution Reaction. *ACS Appl Energy Mater* **2020**, *3* (6), 5597–5609. https://doi.org/10.1021/acsaem.0c00544.

(2) Balqis, F.; Irmawati, Y.; Geng, D.; Nugroho, F. A. A.; Sumboja, A. Nanostructured Ball-Milled Ni–Co–Mn Oxides from Spent Li-Ion Batteries as Electrocatalysts for Oxygen Evolution Reaction. *ACS Appl Nano Mater* **2023**. https://doi.org/10.1021/acsanm.3c02092.

(3) Wang, Z.; Li, S.; Zhang, G.; Yu, X.; Shi, Y.; Zhang, Y.; Xiao, X. Facile Synthesis of FeCoW Oxides: Effects of Amorphous Structure, Electronic Configuration and Catalytic Sites on Water Oxidation. *J Alloys Compd* **2023**, *933*, 167787. https://doi.org/https://doi.org/10.1016/j.jallcom.2022.167787.

(4) Tian, T.; Gao, H.; Zhou, X.; Zheng, L.; Wu, J.; Li, K.; Ding, Y. Study of the Active Sites in Porous Nickel Oxide Nanosheets by Manganese Modulation for Enhanced Oxygen Evolution Catalysis. *ACS Energy Lett* **2018**, *3* (9), 2150–2158. https://doi.org/10.1021/acsenergylett.8b01206.

(5) Zhao, M.; Shen, X.; Zhou, H.; Wang, X.; Wei, Z.; Lv, J.; Zhang, M.; He, G.; Yang, L. Bimetal Mo–Ni/NC as an Effective Electrocatalyst with Accelerated Kinetics for the Oxygen Evolution Reaction. *Journal of Physics and Chemistry of Solids* **2022**, *165* (September 2021), 110651. https://doi.org/10.1016/j.jpcs.2022.110651.

(6) Bo, X.; Li, Y.; Hocking, R. K.; Zhao, C. NiFeCr Hydroxide Holey Nanosheet as Advanced Electrocatalyst for Water Oxidation. *ACS Appl Mater Interfaces* **2017**, *9* (47), 41239–41245. https://doi.org/10.1021/acsami.7b12629.

(7) Ramsundar, R. M.; Debgupta, J.; Pillai, V. K.; Joy, P. A. Co3O4 Nanorods—Efficient Non-Noble Metal Electrocatalyst for Oxygen Evolution at Neutral PH. *Electrocatalysis* **2015**, *6* (4), 331–340. https://doi.org/10.1007/s12678-015-0263-0.

(8) Chen, Z.; Kronawitter, C. X.; Koel, B. E. Facet-Dependent Activity and Stability of Co3O4 Nanocrystals towards the Oxygen Evolution Reaction. *Physical Chemistry Chemical Physics* **2015**, *17* (43), 29387–29393. https://doi.org/10.1039/C5CP02876K.

(9) Wrobel, F.; Park, H.; Sohn, C.; Hsiao, H.-W.; Zuo, J.-M.; Shin, H.; Lee, H. N.; Ganesh, P.; Benali, A.; Kent, P. R. C.; Heinonen, O.; Bhattacharya, A. Doped NiO: The Mottness of a Charge Transfer Insulator. *Phys. Rev. B* **2020**, *101* (19), 195128. https://doi.org/10.1103/PhysRevB.101.195128.
